# Idiosyncratic Reaction with Cytokine Storm Associated with Oxaliplatin

**DOI:** 10.7759/cureus.615

**Published:** 2016-05-19

**Authors:** Vikas Dembla

**Affiliations:** 1 Investigational Cancer Therapeutics, The University of Texas MD Anderson Cancer Center; 2 Hematology-Oncology, Overton Brooks VA Medical Center

**Keywords:** cytokine storm, oxaliplatin, idiosyncratic reaction, fever

## Abstract

Oxaliplatin or irinotecan with 5-fluorouracil form the chemotherapy backbone of any systemic treatment of colorectal cancer. We successfully treated an idiosyncratic reaction to oxaliplatin consisting of severe chills, vomiting, diarrhea, and fever in a patient with metastatic colon cancer.

## Introduction

Here, we report an idiosyncratic reaction to oxaliplatin in a patient with metastatic colon cancer. We also describe the successful management of this reaction, which allowed the patient to undergo further treatment with oxaliplatin. Although platinum hypersensitivity reactions are well documented in literature, they occur most frequently with carboplatin and cisplatin. Oncologists need to anticipate this symptom as part of the constellation of symptoms for a possible idiosyncratic reaction in patients receiving oxaliplatin.

## Case presentation

A 57-year-old man was diagnosed with adenocarcinoma of the sigmoid colon and underwent sigmoid resection. The pathologic analysis showed low grade, moderately differentiated adenocarcinoma, invading through the muscularis propria into the pericolorectal tissues. Margins were negative, the tumor involved 21 of 33 adjacent lymph nodes. Perineural invasion and lymphovascular invasion were also noted. The tumor expressed wild type KRAS. Staging computed tomography (CT) did not reveal any distant metastases. The final pathologic stage was pT3 N2b M0 indicating TNM Stage III-C. Hence, the patient received adjuvant chemotherapy with capecitabine plus oxaliplatin every three weeks for eight cycles (six months total). As a result, he developed Grade 2 peripheral neuropathy, which was treated with supportive therapy of gabapentin and later pregabalin. After completion of adjuvant chemotherapy, there was no evidence of residual cancer per post-treatment CT scans, carcinoembryonic antigen levels (which were normal), or full colonoscopy to the cecum.

However, the patient developed multiple unresectable liver metastases from the colon carcinoma four months after completion of the adjuvant chemotherapy. The patient was treated with various combinations of palliative chemotherapy agents. He first received 5-fluorouracil, leucovorin, and irinotecan (FOLFIRI) plus anti-VEGF therapy with bevacizumab. All therapies this patient received were given per the National Comprehensive Cancer Network (NCCN) guidelines. Upon progression of the cancer, that regimen was followed by FOLFIRI plus anti-EGFR therapy with cetuximab. Upon a second progression, the regimen was switched to regorafenib. Finally, upon a third progression, he received irinotecan plus cetuximab. Oxaliplatin has not yet been readministered in the metastatic setting because of the residual neuropathy from prior oxaliplatin use in the adjuvant setting.

When a fourth progression of disease was confirmed, while the patient was on irinotecan + cetuximab, he still had excellent functioning with Eastern Cooperative Oncology Group performance status of 0. He decided against participating in clinical trials and declined hospice. Approximately three months after the fourth progression of disease, the patient’s neuropathy from oxaliplatin had resolved; hence, he agreed to re-start palliative chemotherapy with capecitabine, oxaliplatin, and bevacizumab given the significant time lapse (more than two years) since he last received those drugs. He tolerated the first cycle of all three agents without any complications, in particular no worsening in neuropathy was reported.

However, right after the second cycle of oxaliplatin, the patient experienced severe chills, vomiting, and diarrhea (bevacizumab was administered prior to oxaliplatin). His vital signs recorded at that time were: temperature 98.1ºF, heart rate 122/minute, blood pressure 102/62 mm Hg, respiratory rate 24/minute, oxygen saturation 96% on room air, he was alert and oriented to person, place, and time but diaphoretic. Assuming these symptoms to be that of a hypersensitivity reaction to either oxaliplatin or bevacizumab, we promptly treated these symptoms with intravenous steroids, diphenhydramine, ondansetron, and meperidine and later with oral loperamide and acetaminophen as needed. The patient required hospitalization for these symptoms. A few hours after admission, he also became febrile with an oral temperature of 102ºF (38.9ºC). Blood and urine cultures were obtained, and he was promptly started on intravenous broad-spectrum antibiotics. He also mentioned that the above symptoms started soon after he ate an unrefrigerated deli meat sandwich that had been made six hours previous, raising the possibility of gastro-enteritis in the differential diagnosis for his acute presentation. The side effects resolved with supportive care in the hospital, and the patient was discharged home on oral antibiotics. Negative culture results were subsequently reported, and all work-up results including fecal studies were negative for an infectious etiology.

Surprisingly, the patient experienced similar symptoms after the completion of the next cycle of capecitabine, bevacizumab, and oxaliplatin leading to treatment similar to what was given previously for those side effects. The side effects resolved after prompt initiation of supportive therapy, and once again, the infectious work-up results were negative. Significantly this time, the patient denied consuming any kind of unrefrigerated meats or food.

Because of the close association of the patient’s acute symptoms with chemotherapy administration and after we eliminated the possible common etiologies such as infections or anaphylaxis, we concluded that these symptoms represented a major cytokine storm–like event. Therefore, after a literature review, it became clear that these symptoms were more representative of an idiosyncratic reaction to oxaliplatin than a reaction to bevacizumab. Hence, this was discussed with the patient and family, and they agreed to an administration of prophylactic intravenous dexamethasone, diphenhydramine, famotidine, and meperidine immediately after the completion of oxaliplatin treatment in addition to routine premedications per standard of care. We also decided to administer the next cycle of chemotherapy in the hospital setting under close observation to prevent any further complications. After the addition of the prophylactic medications after chemotherapy, there was no recurrence of any of the patient’s prior acute adverse effects. Sliding-scale insulin was given to control hyperglycemia secondary to an additional dose of prophylactic dexamethasone. The patient was discharged safely and resumed further chemotherapy with capecitabine, oxaliplatin and bevacizumab on an outpatient basis with the same approach of adding prophylactic medications after each oxaliplatin administration. With this approach, he was able to receive the same chemotherapy regimen for an additional six cycles without any further complications before another progression of cancer was documented. At this time, the patient elected to receive hospice care, given the lack of further active lines of therapy, and he declined to participate in clinical trials.

## Discussion

We determined that our patient suffered from an idiosyncratic reaction consisting of a cytokine storm with acute side effects to oxaliplatin, as similar adverse effects have been described after oxaliplatin use [1-2]. An idiosyncratic reaction is a type B hypersensitivity reaction. These Grade 3 and Grade 4 reactions are rare, occurring in about 1.6% of individuals who receive oxaliplatin [3]. Idiosyncratic drug toxicity may be either non-allergic (metabolic) or immunologic/allergic [4]. The adverse effects described in our case report are most consistent with an immunologic idiosyncratic reaction.

Fever was not an immediate presentation of the reaction in our case, but oncologists need to anticipate fever as part of the constellation of adverse effects of a possible idiosyncratic reaction. Nozawa et al., [5] described a similar case in a 53-year-old female with metastatic rectal cancer who was successfully rechallenged with oxaliplatin by using prophylactic antiemetics, corticosteroids, and antihistamines twice (before and after oxaliplatin), and as a result, the patient was able to receive oxaliplatin for five cycles. Oxaliplatin is not known to have any adverse effects on the cytochrome P450 transporter system [6]. A review of our patient’s medication list showed that his risk of interactions was very low (he was taking losartan, amlodipine, and tramadol for use as needed. He denied drinking grapefruit juice or using any over the counter herbal medications.

## Conclusions

Idiosyncratic reaction is a known but uncommon adverse effect of oxaliplatin, but its incidence is increasing with more widespread use of oxaliplatin [3]. In contrast, platinum hypersensitivity to carboplatin and cisplatin is well described in the literature, with rates varying from 1% to as high as 35% depending upon the severity and grade, and the risk of reaction increases with each subsequent cycle [7]. We were successfully able to rechallenge with oxaliplatin in this patient and prevent idiosyncratic reaction with the addition of prophylactic medications after oxaliplatin infusion, after which he was able to receive oxaliplatin for an additional six cycles. Our outcome in this case is consistent with those published in the literature [1-2,5]. If patients with metastatic colon cancer suffer from an idiosyncratic reaction to oxaliplatin, there is an option to switch to irinotecan in the chemotherapy backbone. However, in adjuvant treatment of colorectal cancer, irinotecan is not approved for use, and hence, substitution of oxaliplatin by irinotecan is not an option. To manage an idiosyncratic reaction to oxaliplatin in a patient receiving adjuvant chemotherapy for colorectal cancer either with 5-fluorouracil and leucovorin (as FOLFOX) or with capecitabine (as CAPOX), one option that could be considered is an intensive prophylactic medication approach as we used in our case—intravenous dexamethasone, diphenhydramine, famotidine, and meperidine given immediately after the oxaliplatin infusion. The optimum desensitization protocol for oxaliplatin administration in terms of efficacy and tolerability still needs to be defined.
